# Effectiveness of behavioural graded activity compared with physiotherapy treatment in chronic neck pain: design of a randomised clinical trial [ISRCTN88733332]

**DOI:** 10.1186/1471-2474-5-34

**Published:** 2004-10-06

**Authors:** Frieke Vonk, Arianne P Verhagen, Mario Geilen, Cees J Vos, Bart W Koes

**Affiliations:** 1 Department of General Practice, Erasmus MC, University Medical Centre Rotterdam, the Netherlands; 2 Department of Rheumatic diseases and Chronic pain, Hoensbroek Rehabilitation Centre, the Netherlands

## Abstract

**Background:**

Chronic neck pain is a common complaint in the Netherlands with a point prevalence of 14.3%. Patients with chronic neck pain are often referred to a physiotherapist and, although many treatments are available, it remains unclear which type of treatment is to be preferred.

The objective of this article is to present the design of a randomised clinical trial, Ephysion, which examines the clinical and cost effectiveness of behavioural graded activity compared with a physiotherapy treatment for patients with chronic non-specific neck pain.

**Methods:**

Eligible patients with non-specific neck pain persisting longer than 3 months will be randomly allocated to either the behavioural graded activity programme or to the physiotherapy treatment. The graded activity programme is based on an operant approach, which uses a time-contingent method to increase the patient's activity level. This treatment is compared with physiotherapy treatment using a pain-contingent method.

Primary treatment outcome is the patient's global perceived effect concerning recovery from the complaint. Global perceived effect on daily functioning is also explored as primary outcome to establish the impact of treatment on daily activity. Direct and indirect costs will also be assessed. Secondary outcomes include the patient's main complaints, pain intensity, medical consumption, functional status, quality of life, and psychological variables. Recruitment of patients will take place up to the end of the year 2004 and follow-up measurement will continue until end 2005.

## Background

### Prevalence and incidence

Neck pain is a common complaint that causes substantial morbidity in western countries with a reported prevalence ranging from 9.5 to 22% [1,2]. Of all musculoskeletal pains in the Netherlands, neck pain is one of the three most reported with a point prevalence of 21%; it is more often reported by women than men [3]. In 1996 total related costs were estimated to be US $686.2 million, which is about 1% of the total Dutch health care expenditures [4]. Most neck complaints are continuous or recurrent [3]. When the neck pain persists for more than 3 months it is defined as chronic, and the related prevalence is 14.3% [3,5]. Although the prevalence of neck pain is stable over different age groups, the incidence of chronic neck pain increases with age [3,6].

There are many potential causes of neck pain, but mostly no specific underlying pathology is found so that it is designated as non-specific [7]. Although not a life- threatening disease, neck pain can negatively affect patients' quality of life, cause pain and stiffness, and may result in substantial medical consumption, absenteeism and disability [4,8].

In the Netherlands, patients with neck pain are often referred for physiotherapy. Moreover, physiotherapy accounted for 84% of the total direct medical neck pain costs in 1996 [4]. Although physiotherapists can apply various treatments, no formal guidelines are yet available.

### Treatment models

Two treatment models have been described in the literature, both of which are applicable within the field of physiotherapy. The first, a biomedical model, considers pain to be a sign of physiological damages and treatment according to this model aims to remove the pathologic condition so that the pain will no longer occur [9,10]. Moreover, treatment is guided by the amount of pain a patient experiences, leading to a pain-contingent approach [11]. According to the second, a biopsychosocial model, pain is not necessarily caused by underlying pathology or impairment but can persist long after the initial pathology has healed; psychological and social factors may be important in the development and maintenance of complaints [12,13]. According to the principles of this biopsychosocial model, behavioural therapies assume that maladaptive behaviours are learned and, therefore, can be modified through new learning experiences [10,14]. Three different approaches are known: respondent, operant and, cognitive behavioural therapy [9,15,16]. The present study mainly employs an operant behavioural approach, as described by Fordyce and applied by Lindström et al [11,17]. According to this approach, the treatment focuses on decreasing pain behaviour (operants) and increasing healthy behaviour, and consists of behavioural graded activity on a time-contingent basis [11,18].

### Available evidence

Many conservative physiotherapeutic treatments are available for treating neck pain, but there is insufficient evidence to allow to conclude that one type of treatment is more effective then others [19,20].

In a review on chronic pain, operant behavioural therapy was found to be beneficial to waiting list control groups on outcomes such as pain experience, mood effect other than depression, social role, and for the expression of pain behaviour [21]. Compared to other treatments, operant behavioural therapy is only beneficial for the expression of pain behaviour and role functioning [21]. Another review showed little evidence that biopsychosocial multidisciplinary rehabilitation is more effective than other rehabilitation methods for neck and shoulder pain, but the authors found only two relevant studies that satisfied the criteria for their review [22]. When examining the effectiveness of behavioural treatment for chronic pain another difficulty is that no standard protocol exists for the application of these treatments. As a result, a wide range of techniques described in the literature has been labelled as behavioural [23].

In summary, it remains unclear which type of conservative, including behavioural, treatment is to be preferred in the management of chronic neck pain. Therefore, this study, Ephysion (Effectiveness physiotherapy in neck pain), aims to evaluate the clinical and cost effectiveness of an operant behavioural programme (i.e. behavioural graded activity) compared with a physiotherapy treatment in patients with chronic non-specific neck pain. In addition, we aim to identify subgroups of patients who benefit most from one of the two treatments, and to identify the most important determinants for recovery from chronic non-specific neck pain.

### Why a design article

Because a biased study design can produce incorrect conclusions, the design of a trial should be carefully examined before adopting its conclusions [24]. A design article allows to examine the design objectively without being influenced by the study results, to check any resulting articles for protocol deviations, and may also reduce the temptation to search for associations during data analysis rather then presenting hypotheses in advance [25]. Further, a published protocol informs others about which studies are in process thus reducing duplication of research effort [25]. Finally, a design article prevents publication bias in the case that future articles are not published, because study results can be retrieved from the author and the study can therefore still be included in future reviews [25,26].

## Methods

### Study design

A randomised clinical trial (RCT) has been designed to assess the effectiveness of behavioural graded activity compared with physiotherapy treatment in patients with chronic non-specific neck pain. The study design has been approved by the Medical Ethics Technical Commission of the Erasmus MC, University Medical Centre in Rotterdam and is in compliance with the Helsinki Declaration.

### Selection of patients and informed consent

Forty general practitioners (GP) in region West Brabant in the Netherlands will select the patients. Patients are eligible if they are aged between 18 and 70 years old, have suffered from neck pain for over three months, and have an adequate knowledge of the Dutch language. Excluded are patients diagnosed with a specific disorder (e.g. a slipped disc, a tumour or a lesion in the cervical spine), those who have had physical/manual therapy during the previous six months, those with a chronic disease (e.g. rheumatoid arthritis or coronary artery disease), or those who have to undergo surgery in the near future. Eligible patients will receive an information leaflet from their GP and the GP then informs the research department.

Thereafter, the research assistant contacts the patient, provides additional information about the implications of participation, re-checks the eligibility of the patient, and completes the informed consent procedure.

### Sample size

The sample size for this study is calculated according to the global perceived effect (GPE). Based on previous studies, a 20% difference in GPE is expected after completion of either treatment (9 weeks) and is considered to be clinically relevant; 160 patients are needed to detect this difference. In this calculation a power (1 - β) of 80% is taken into account. Thus, the inclusion of 80 patients per treatment group is planned.

### Randomisation

An independent examiner using a computer-generated randomisation schema performs randomisation. To prevent unequal distribution, patients are pre-stratified based on three important prognostic factors: gender, age and the severity of the complaint, which are recorded at baseline [27]. Further, unequal group sizes are prevented by using a 6-block randomisation that equalizes allocation to the two treatment groups per stratum after every sixth patient [28]. After randomisation, patients choose a physiotherapist within the allocated treatment group. Then, to ensure that the treatment starts as soon as possible, the research assistant makes the first appointment for treatment.

### Blinding

Patients are told to receive physiotherapy but are blinded to allocation of the two treatments; the content of the treatments is not described in the information leaflet. This enhances the quality of the study, because the patients themselves measure the effect of treatment. GPs are also blinded for allocation to prevent accidentally informing the patients of the allocated treatment. The physiotherapists are not blinded for allocation, but the physiotherapists from each treatment group are kept strictly separate and are not involved in the outcome measurement. Finally, the primary investigator is blinded for patients' allocation but the research assistant is not; neither is involved in the outcome measurement.

### Physiotherapists and Interventions

After receiving written information, 34 physiotherapists in region West Brabant will participate in either the physiotherapy treatment (PT) or the graded activity programme (GAP). To optimise the contrast between the two treatments, both groups are strictly separated throughout the study. The PT group consists of 16 physiotherapists and the GAP group of 18 physiotherapists. The PT physiotherapists participate in a meeting to standardize the physiotherapy treatment. The GAP physiotherapists are instructed on the behavioural graded activity approach during a two-day theoretical and practical training course.

Both interventions are performed in an outpatient setting. A maximum of 18 treatments per patient is set and each treatment takes about 30 minutes, which is in accordance with medical insurance policy in the Netherlands. Before treatment starts, physiotherapists receive a completed questionnaire about the patient's main complaints [29]; this questionnaire reveals the three daily activities which are considered the most important complaints to the patient. Physiotherapists can use these three activities in the process of formulating the patient's primary therapy aim. In both treatments, the physiotherapist starts with a physical examination of the patient and an anamnesis. Then an individually tailored program will be applied and the process recorded after each treatment session using a specially designed form.

#### The physiotherapy treatment

The content of the physiotherapy treatment is decided by consensus among the participating PT physiotherapists. Treatment is according to a biomedical model, which implies guidance based on the amount and severity of pain that the patient experiences.

By consensus, the physiotherapy treatment is divided into the patient's primary therapy aim, three general treatment goals, and several techniques to attain those goals. The primary therapy aim is defined as the result the patient wants to achieve by the end of therapy. A general treatment goal is a goal for each single treatment and could, therefore, differ per treatment session. Table 1 shows the three general treatment goals, together with the techniques physiotherapists can choose to attain them. In daily practice a broad spectrum of treatment techniques are available, but in this study the techniques to be used consist of physiotherapy techniques with a strong focus on exercises. Moreover, manipulative techniques, acupuncture and other (alternative) techniques are excluded, as are physiotherapeutic applications such as ultrasound or diathermy.

#### Behavioural graded activity

An operant approach was the basis of the behavioural graded activity programme as used in this study. The treatment is according to a biopsychosocial model, which implies that it is guided by the patients' functional abilities and that time-contingent methods are used to increase the activity level of the patient [11]. The behavioural graded activity programme has three phases; a baseline phase, a treatment phase, and a generalization phase. These phases are not bound to strict time limits but can gradually merge into each other.

Before starting the baseline phase, the treatment vision and the patient's ideas about pain and its causes are discussed. The development and maintenance of pain will be explained and patients are reassured that it is safe to move and to increase their level of activity [11,13,30]. Both are explained by means of a pain model, which has been derived from the fear-avoiding-model of Vlaeyen et al. [13]. Thereafter primary therapy aims are formulated based on the patient's main complaints, which are described as three daily activities and were revealed in the baseline questionnaire. For each of these activities, a baseline level of intensity is determined based on a pain-contingent measure. This means that patients perform each activity at least three times, each time until they have to stop because of their pain. Afterwards, patient and physiotherapist together set a start quota and time-contingent treatment quotas for each activity. The quotas will be based on the patient's mean baseline scores, primary therapy aims [17], and on the behaviour that can be derived from the baseline measure. If necessary, facilitating disorder-oriented exercises can be added to the treatment as preparation for the activities that were pointed out as main complaints. The same approach as used for the main complaint is used for these exercises.

During the treatment phase, patients systematically increase the time-contingent quotas to enable them to reach their personal aims within a pre-set therapy time period. To ensure a successful experience during the first exercise, the start quota is below the mean baseline score. The pre-set exercise quotas have to be strictly followed; neither over-performance nor under-performance is allowed. During this phase the patient has to practice at home and document every activity or exercise on a performance chart. These charts will be discussed in the following treatment session and achievements will be reinforced while disregarding pain behaviours. Positive reinforcements of healthy behaviour and the patient's experiences of success are considered to be important to enhance the patient's motivations.

The generalization phase takes place at the end of the treatment phase. In this phase generalization of learned behaviour and management of relapses will be discussed.

### Outcome measurement

Baseline questionnaires are sent after inclusion, which is as soon as possible after patients have consulted their GP. Outcome of intervention will be assessed at 4 and 9 weeks after randomisation; however, if the treatment is not finished at 9 weeks, the patients will receive an additional questionnaire (Ts) after finishing the treatment. Follow-up assessments are planned at 26 and 52 weeks after randomisation.

All outcome measures are reported by means of mailed questionnaires. Table 2 presents the outcome variables, the instruments used and the moments at which they are measured.

Primary treatment outcome of this study is the global perceived effect, which is used to assess recovery from the complaint [31]. In addition, the global perceived effect in daily functioning was explored in order to also establish impact of treatment on daily activity. Both treatment outcomes (recovery of complaint and functioning in daily activity), are assessed on a 7-point Likert-scale, ranging from completely recovered (1) to worse than ever (7).

Costs are measured using a combination of questionnaires to collect data on direct medical costs (e.g. the amount of received treatment and additional therapy received), and indirect costs due to sick leave and disability.

Secondary outcome measures include main complaints, pain intensity, medical consumption, coping, functional status, quality of life, and psychological variables. Prognostic factors are measured including demographic variables, the baseline variables and the psychological variables (table 2).

### Analyses

Descriptive statistics will be used to examine comparability of baseline data between PT and GAP, and to check if randomisation was successful. Before this analysis, decisions about differences considered to be clinically relevant are made and, if necessary, adjustment will be made for these differences in multivariate analysis. Further, all outcome data will be screened for normality and, if necessary, logarithmic transformations or non-parametric methods of analysis will be applied.

The first aim is to evaluate the clinical and cost effectiveness of GAP compared to PT. Clinical effectiveness will be examined with a Student's t-test (continuous), a Chi-square test (dichotomised) or a Wilcoxon test (not normally distributed) according to the intention-to-treat principle. This means that patients will be analysed in the treatment group to which they are randomly allocated. For missing data, imputation techniques will be used. When the dropout rate is 10% or more, or loss to follow-up is 20% or more, per-protocol analysis will be performed. The results on primary outcome will be dichotomised into improved versus not improved. Improved implies completely recovered and much improved, whereas not recovered implies slightly improved, not changed, slightly worsened, much worsened, and worse than ever [31].

Cost effectiveness will be calculated from a societal perspective. Costs (direct as well as indirect) will be related to the treatment effects, based on the primary outcome measure, by calculating cost-effectiveness ratios.

The second aim is to identify subgroups of patients that benefit most from one of the two treatments. The following subgroups will be investigated: duration and severity of the complaint, depression, and fear of movement.

The third aim is to identify important variables for recovery. For this purpose multivariate analysis will be performed to investigate the influence of prognostic variables and patient characteristics on the outcome. Separate analyses will be conducted to investigate prognostic factors for short-term (3 months) and long-term (12 months) recovery.

## Discussion

This study is designed to evaluate the clinical and cost effectiveness of a behavioural graded activity programme compared with a physiotherapy treatment in patients with chronic non-specific neck pain. Since physiotherapists perform both treatments in this study, contrast between the two treatments is a very important issue. There are contrasts both in the composition of the treatment and the way the physiotherapists approach the patient. With regard to the composition, the graded activity programme (GAP) starts with a systematically performed baseline measurement; this is in contrast to the physiotherapy treatment (PT), where treatment is based on history taking and physical examination. In GAP quotas are set based on the patient's behaviour, whereas in PT they are set based on pain levels and training principles. After quotas are set GAP uses a time-contingent treatment approach, which involves a pre-set systematic increase in activities. In contrast, PT uses a pain-contingent approach, which means that treatment is adapted to the patient's reaction to previous treatment sessions. Furthermore, GAP uses a hands-off approach, whereas PT may contain hands-on techniques, such as massage, traction etc (Table 1).

This study addresses an important question because chronic neck pain is a common complaint and it remains unclear which type of physiotherapeutic treatment is most effective. Recruitment of patients will take place until up to the end of 2004; follow-up measurement will continue up to end 2005.

## Competing interests

The authors declare that they have no competing interests.

## Authors' contributions

APV and BWK conceived the study, developed the design of the randomised clinical trial and participated in writing the article. MG is an expert in the field of graded activity and contributed to the content of the article. CJV advised on the content of the article. FV conducts the research, participated in the completion of the study design and wrote the article. All authors have read and approved the final manuscript.

## Pre-publication history

The pre-publication history for this paper can be accessed here:



## Figures and Tables

**Table 1:** Treatment goals and techniques that can be used to reach the primary treatment aim.

Treatment goals	Techniques
relaxation and preperation for exercise	- Massage
	- Relaxation exercise
	- Thoracal treatment up to thoracal 9.
	- Localized 3-d mobilization within physiological boundary of the joint capsule
	- Mobilization in al directions within physiological boundaries.
	- Traction within physiological boundaries.
	- Techniques of Mulliken excluding manipulation
	- Techniques of McKenzie excluding manipulation
Education	Can take place at the same time as the first treatment target. Education includes patient reassurance; explanation of (physiological) load and capability of carrying a load; and encouragement of physical activity
Exercise	- Passive exercise, guided active exercise, and active exercise
	- Exercise at the physiotherapist
	- Assign homework

**Table 2:** Overview of variables measured in this study

**Variable**	**Time Measured**						**Range of unit**
	**T0**	**T4**	**T9**	**Ts**	**T26**	**T52**	

*Inclusion and exclusion variables*	x						
**Demographic variables**	x						
**Baseline variables**							
Specific complaint characteristics	x						
Experience of the neck complaint and functioning in daily activities	x						1–7 (Likert scale)
Co-morbidity	x						
Additional complaints	x						
**Primary outcome**							
'Global perceived effect' (neck complaint and functioning in daily activities) [31]		x	x	x	x	x	1–7 (Likert scale)
**Secondary outcomes**							
Main complaint [29]	x	x	x	x	x	x	0–10 (Likert scale)
Pain (VAS) [31]	x	x	x	x	x	x	
Medical consumption	x	x	x	x	x	x	Dose per day
Coping with Multi-dimensional pain (MPI) Part I-II [32]	x		x		x	x	0–6 (Likert scale)
Activity (MPI, part III)	x	x	x	x	x	x	0–6 (likert scale)
Specific functional status (NDI) [33]	x	x	x	x	x	x	
Quality of life (SF-36) [34, 35]	x		x		x	x	
(EQ-5d) [35, 36]	x	x	x	x	x	x	
Work activities	x		x		x	x	Hours/week
Satisfaction about treatment		x	x	x	x		1–5 (Likert scale)
Compliance with treatment exercise		x	x	x	x	x	Number and time per week
Additional treatments		x	x	x	x	x	Discipline and number of treatments
Side-effects		x	x	x	x	x	Yes – No and any additional elucidation
**Psychological (prognostic) variables**							
Fear of movement (TSK) [37]	x				x	x	1–4 (likert scale)
Catastrophizing (PCS) [38]	x		x		x		1–5 (likert scale)
Depression (CES-D) [39]	x		x		x		1–4 (likert scale)
Self-efficacy (PSEQ) [40]	x	x	x	x	x	x	10–100% (very unsure – very sure)
Stages of change (PSOCQ) [41]	x		x				1–5 (likert scale)

Note: T_0 _= baseline measurement, T_4_, T_9_, (T_S_), T_26_, T_52 _are follow-up measurements at 4, 9, 26 and 52 weeks, respectively, after randomisation. Ts was received at the end of treatment, when treatment lasted longer than 9 weeks. *MPI*, Multidimensional Pain inventory; *NDI*, Neck Disability Index; *SF-36*, Short Form 36; *EQ-5d*, Euroquol 5-Dutch language version; *TSK*, Tampa Scale for Kinesiophobia; *PCS*, Pain Catastrophizing Scale; *CES-D*, Centre for Epidemiologic Studies – Depression; *PSEQ*, Pain Self-Efficacy Scale; *PSOCQ*, Pain Stages Of Change Questionnaire.
